# Role of Oxidative Stress in Vascular Low-Grade Inflammation Initiation Due to Acute Salt Loading in Young Healthy Individuals

**DOI:** 10.3390/antiox11030444

**Published:** 2022-02-23

**Authors:** Ana Knezović, Nikolina Kolobarić, Ines Drenjančević, Zrinka Mihaljević, Petar Šušnjara, Ivana Jukić, Marko Stupin, Aleksandar Kibel, Saška Marczi, Martina Mihalj, Ana Stupin

**Affiliations:** 1 Community Health Center Osijek-Baranja County, Park Kralja P. Krešimira IV/6, 31000 Osijek, Croatia; anaknezovic.os@gmail.com; 2 Department of Physiology and Immunology, Faculty of Medicine Osijek, Josip Juraj University of Osijek, J. Huttlera 4, 31000 Osijek, Croatia; nbdujmusic@mefos.hr (N.K.); ines.drenjancevic@mefos.hr (I.D.); zrinka.mihaljevic@mefos.hr (Z.M.); psusnjara@mefos.hr (P.Š.); ivana.jukic@mefos.hr (I.J.); mstupin@mefos.hr (M.S.); alekibel@mefos.hr (A.K.); 3 Scientific Center of Excellence for Personalized Health Care, Josip Juraj Strossmayer University of Osijek, Trg Svetog Trojstva 3, 31000 Osijek, Croatia; 4 Department for Cardiovascular Disease, University Hospital Osijek, J. Huttlera 4, 31000 Osijek, Croatia; 5 Laboratory of Molecular and HLA Diagnostics, Clinical Institute of Transfusion Medicine, University Hospital Osijek, J. Huttlera 4, 31000 Osijek, Croatia; saska.marczi@kbco.hr; 6 Department of Medical Chemistry, Biochemistry and Clinical Chemistry, Faculty of Medicine Osijek, Josip Juraj University of Osijek, J. Huttlera 4, 31000 Osijek, Croatia; 7 Department of Dermatology and Venereology, Osijek University Hospital, J. Huttlera 4, 31000 Osijek, Croatia

**Keywords:** high-salt diet, inflammation, oxidative stress, cytokines, Th17 cells, regulatory T cells, cell adhesion molecules, endothelium

## Abstract

This study aimed to investigate the effect of 7-day high-salt (HS) and the specific role of oxidative stress on vascular low-grade inflammation initiation in young salt-resistant healthy individuals. 30 young healthy individuals adhered to a 7-day low-salt (LS) diet (3.5 g salt/day), followed by a 7-day high-salt (HS) diet (~14.7 g salt/day) protocol. Pro- and anti-inflammatory cytokines, frequencies of peripheral blood Th17 and Treg cells, Th17/Treg ratio, enzymes SGK1, and p38/MAP kinase, as well as biomarkers of endothelial activation and oxidative stress, were measured before and after the 7-day HS diet protocol. Short-term HS diet significantly increased serum level of pro-inflammatory cytokines INF-γ, TNF-α, IL-9, and IL-17A levels, but also of anti-inflammatory cytokines IL-10 and TGF-β1. Relative amount of total SGK1 significantly increased, following the 7-day HS diet. Increased oxidative stress level, following HS diet, was negatively associated with the frequency of Treg cells. The increase in relative amount of total SGK1 in peripheral mononuclear cells following 7-day HS diet suggests lymphocyte (re)activation, in response to HS intake, resulting in enhanced production of pro-inflammatory (IL-17, INF-γ), but also anti-inflammatory cytokines (IL-10 and TGF-β1). Increased oxidative stress, due to HS loading, alters immune regulatory mechanisms, presumably via effects on Treg cells.

## 1. Introduction

Excessive salt intake via daily diet has been well-associated with development and progression of various cardiometabolic diseases, e.g., hypertension. Pathogenesis of these diseases encompasses complex interactions of several factors including sex, age, renin-angiotensin system (RAS), autonomic nervous system, endothelial function, and oxidative balance [1,2], of which, some are affected by the high-salt (HS) diet. Even in healthy individuals, HS loading leads to endothelial dysfunction [3], a hallmark of cardiometabolic diseases, characterized by impaired vascular reactivity to various stimuli, prior to the changes in blood pressure (BP), and independent of fluid status or composition of the body changes [4,5]. In particular, decreased bioavailability of nitric oxide (NO), due to an increased oxidative stress level, was detected as one of a key mechanism that underlies impaired endothelium-dependent vasodilation in healthy individuals on a HS diet [6,7]. The role of oxidative stress in impaired vasodilation was supported by the fact that localized micro-dialysis of vitamin C has completely restored NO-mediated arteriolar dilation in humans on a HS diet [8]. Moreover, our recent study showed that enhancing of antioxidant defense by peroral intake of vitamin C and E, concomitantly with a 7-day HS diet, prevented increase in oxidative stress and impairment of microvascular endothelial function in young healthy individuals on HS diet [9].

The immune system is known to play an important role in the development and progression of endothelial dysfunction underlying hypertension, and the excessive salt intake might also be directly associated with altered regulation of the immune system, leading to aberrant immune responses [10]. Endothelial dysfunction is characterized by endothelial activation, resulting in the vascular inflammation, caused by leukocytes chemoattraction and transmigration to the vascular wall [11]. Studies in experimental animals reported an overexpression of cell adhesion molecules (e.g., ICAM-1, VCAM-1, and E-selectin), following even short HS loading, indicating an early development of endothelial–leukocyte interactions [12,13,14]. In vitro studies demonstrated the enhanced polarization of T helper lymphocytes to the T helper 17 (Th17) lineage, when these cells were primed under HS conditions [15,16]. The mechanism underlying Th17 cells generation involves activation of the p38 mitogen activated protein (p38/MAP) kinase pathway and downstream serum- and glucocorticoid-induced kinase 1 (SGK1) [16,17], which may be altered by stressors, such as hyperosmolarity, due to a HS diet. In addition, polarization and expansion of IL-17-secreting T cells was also linked to the activity of local RAS in the kidneys during subacute exposure of rats to excessive dietary salt following recovery from acute kidney injury [15,16].

Interestingly, there are evidences that HS intake can cause abnormal interactions between leukocytes and endothelial cells [18], induce pro-inflammatory cytokines production [19], and modulate immune cell (mainly macrophages and T cells) [20,21] functions in healthy humans, but clinical studies on the earliest effects of salt loading on the immune system and how oxidative stress relates to these effects are scarce. For example, elevation in extracellular sodium concentration has led to CD14^++^CD16^+^ cells expansion via a ROS-dependent manner, as revealed in vitro study [22]. A total of 50 days of a HS diet (12 g salt/day) resulted in a higher number of peripheral monocytes in healthy subjects, while a lower salt intake (6 and 9 g of salt/day) resulted in a reduced production of IL-6 and IL-23 and enhanced production of the anti-inflammatory cytokine IL-10 [19]. In another human study, a short-term HS diet induced expansion of CD14^++^CD16^+^ “intermediate” monocytes, which is associated with target organ inflammation in humans [22]. Our research group also demonstrated that a 7-day HS diet substantially altered the peripheral blood leukocytes’ phenotype and dynamics, independently of BP changes in healthy individuals [21]. On the other hand, very little is known from human studies about HS diet induced changes of the adaptive immune responses, especially in terms of Th17/Treg interplay. Luo and colleagues demonstrated perturbations of peripheral IL-17 and Foxp3-expressing CD4^+^ T cell compartments during the 7-days HS diet and adjacent low-salt (LS) diet period in healthy male volunteers; however, detailed immunophenotyping of peripheral Th17 and Treg lymphocytes, following acute salt loading, is still missing [20]. Taken together, accumulating evidence supports the interaction between salt intake and immunity, intertwined with changes in oxidative stress-antioxidative balance, but a profound understanding of such a relationship is still in its infancy.

Thus, the present study aimed to investigate the effect of a 7-day HS diet on development of vascular low-grade inflammation in young healthy individuals by measuring (1) the systemic level of pro-/anti-inflammatory mediators, (2) the biomarkers of endothelial activation, (3) the frequencies of peripheral regulatory (Treg), and T helper 17 (Th17) lymphocytes, as well as (4) the enzymes involved in their activation and differentiation (SGK1 and p38/MAP kinase). An additional goal was to test the role of increased oxidative stress level, due to HS loading, on modulation of measured potential key players involved in vascular low-grade inflammation initiation in young healthy individuals.

## 2. Materials and Methods

### 2.1. Study Population

The present study involved thirty young healthy individuals of both sexes (15 women and 15 men), with ages ranging from 18–29 years. Participants were recruited at the Faculty of Medicine Josip Juraj Strossmayer University of Osijek, Osijek, Croatia. Obesity [body mass index (BMI) > 30 kg/m^2^], hypertension, hyperlipidemia, renal impairment, diabetes, coronary artery, peripheral artery, and cerebrovascular disease were considered as exclusion criteria for participation in the present study. Exclusion criteria were also taking of oral contraceptives, anti-inflammatory non-steroidal drugs, antihypertensive agents, steroids, or any other drugs/agents that could affect the endothelium. Women were assigned to the study protocol in different phases of their menstrual cycle (randomized), in order to exclude the potential effect of sex hormones fluctuation during menstrual cycle on immune response and endothelial function. Informed consent was obtained from all participants involved in the study. The present study included procedures and protocols that adhered to the standards set by the latest revision of the Declaration of Helsinki. The study was approved by the Ethical Committee of the Faculty of Medicine Josip Juraj Strossmayer University of Osijek, Osijek, Croatia (Cl: 602-04/15-08/08; No: 2158-61-07-15-68) and is also a part of clinical trial investigating effects of HS diet on microvascular endothelial function in healthy individuals, registered at ClinicalTrials.gov (ID NCT02727426 Dietary Salt and Microvascular Function).

### 2.2. Study Protocol

This was a controlled clinical experiment, lasting for 14 days, and the study intervention involved dietary salt perturbation. In order to normalize basal dietary salt intake, all subjects completed 7 days of run-in diet, during which all subjects maintained the low-salt (LS) diet, which implied intake of 3.75 g of salt per day, according to the DASH eating plan (DASH eating plan, US Department of Health and Human Services, 2006). For the next 7 days of the HS diet, all subjects were taking approximately 14.7 g of salt per day, i.e., DASH diet (3.75 g of salt per day) was supplemented with additional 11.2 g of salt per day (in a form of a commercially available kitchen salt powder). Each subject had two repeated measurements of all further described procedures and parameters, with the first measurement following a 7-day LS diet (LS condition) and second measurement following 7-day HS diet (HS condition). All the measurements were performed in the morning on an empty stomach. Data were collected in the Laboratory for Clinical and Sport Physiology, Department of Physiology and Immunology at Faculty of Medicine Josip Juraj Strossmayer University of Osijek, Osijek, Croatia.

### 2.3. 24-h Urine Samples Analysis

To assess compliance to given dietary protocols (LS and HS), each subject collected urine during the last 24-h of the LS and HS diet, according to the given instructions. The 24-h urine was analyzed for sodium, potassium, urea, creatinine coefficient, protein, and albumin concentration. A 24-h urinary sodium excretion was used for the assessment of daily salt intake using appropriate formula [1 g salt (NaCl) = 393.4 mg Na = 17.1 mmol Na]. The 24-h urine samples were analyzed at the Clinical Institute of Laboratory Diagnostics, University Hospital Osijek, Osijek, Croatia.

### 2.4. Anthropometric and Blood Pressure Measurement

Subjects body mass index (BMI) was calculated from measured height (m) and weight (kg), while waist-to-hip ratio (WHR) was calculated from measured waist and hip circumference. Arterial BP was measured at each study visit, using an automated oscillometric sphygmomanometer (OMRON M3, OMRON Healthcare Inc., Osaka, Japan). The final BP value was a median of three consecutive measurements.

### 2.5. Venous Blood Sampling and Analysis

A venous blood sample was taken from each subject at both study visits. Blood samples were immediately analyzed for plasma electrolytes (sodium, potassium, and calcium), creatinine, urea, high sensitivity C reactive protein (hsCRP), and full blood count, using standard laboratory methods and operating protocols at the Clinical Institute of Laboratory Diagnostics, University Hospital Osijek, Osijek, Croatia.

### 2.6. Serum Protein Concentration of Pro- and Anti-Inflammatory Cytokines, C3a Complement Component, Soluble Cell Adhesion Molecules, and Endoglin Assay

For this set of experiments, serum was separated from venous blood samples and stored in a refrigerator at −80 °C, until the experiments were performed. Serum protein concentrations of (a) pro-inflammatory cytokines: interferon gamma (INFγ), interleukin (IL-6), tumor necrosis factor alpha (TNF-α), interleukin 9 (IL-9), interleukin 23 (IL-23), and interleukin 17A (IL-17A); (b) anti-inflammatory (and immunomodulatory) cytokines: interleukin 10 (IL-10), interleukin 21 (IL-21), interleukin 22 (IL-22), and transforming growth factor beta (TGF-β1); (c) C3a complement component; (d) soluble cell adhesion molecules: soluble intercellular adhesion molecule 1 (sICAM-1), soluble vascular cell adhesion molecule 1 (sVCAM-1), and E-selectin (CD-62E); and (e) endoglin were measured with panel for multiplex protein quantitation (Invitrogen ProcartaPlex), using the Luminex 200 instrument platform, according to the protocol described in an earlier paper of our research group [23]. Experiments were done in the Laboratory for Immunology and Allergology Diagnostics, Osijek University Hospital, Osijek, Croatia.

### 2.7. Peripheral Blood Mononuclear Cells (PBMCs) Isolation, Cryopreservation, Thawing, and Cultivation

Peripheral blood mononuclear cells (PBMCs) were isolated from whole blood samples by density gradient centrifugation with Ficoll-Paque^®^ PLUS centrifugation media (GE Healthcare Bio-Sciences AB, Uppsala, Sweden). All procedures and protocols used for PBMCs isolation, cryopreservation, thawing, and cultivation were previously described, in detail, in a previous paper of our research group [24].

### 2.8. Regulatory T Lymphocytes (Treg) and Helper T Lymphocytes (Th17) Frequencies Assay and Th17/Treg Ratio

Flow cytometry method was used for immunophenotyping of regulatory T lymphocytes (Treg) and helper T lymphocytes (Th17), according to the protocol described, in detail, in an earlier paper of our research group [24]. Standard recommended controls for flow cytometry were included in our experiments, in order to accurately recognize positive signals from background and nonspecific immune reaction. The compensation matrix was calculated using appropriate, commercially available compensation beads (BD Biosciences, Becton, Dickinson and Company, Franklin Lakes, NJ, USA). BD FACSCanto II cytometer (FACSCanto II, Becton Dickinson, San Jose, CA, USA) was used for measuring stained samples, and FlowLogic software (Inivai Technologies, Mentone, Australia) was used for data analysis and visualization.

**Figure 1:**
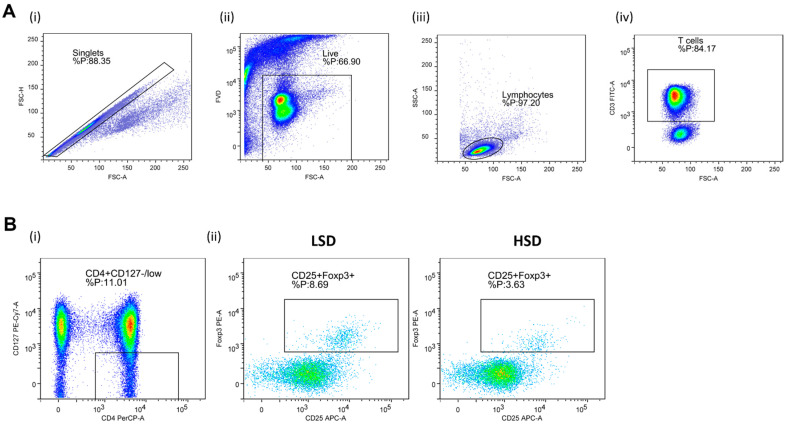
Gating strategy for the assessment of peripheral blood regulatory T cells (Treg) by flow cytometry. Panel (**A**) shows representative dot plots illustrating gating strategy, including exclusion of doublets using forward scatter area (FSC-A) versus forward scatter width (FSC-W) analysis (**A**-**i**), gating on live cells negative for aminereactive fixable viability dye (**A**-**ii**), lymphocytes (**A**-**iii**), and CD3^+^ T cells (**A**-**iv**). Next, total CD25 and Foxp3, expressing T cells among CD4^+^CD127 low population, were analyzed (Panel (**B**)). (**B**-**i**) shows representative gating strategy, (**B**-**ii**) representative samples from LS and HS protocol. FlowLogic software was used for data analysis and illustration.

Regulatory T Lymphocytes (Treg). In order to determine expression of Treg specific cell surface antigens, the following antibody mixture was used: CD3 FITC (clone: OKT3, eBioscienceTM, Affymetrix by Thermo Fisher Scientific, CA, USA), CD4 PerCP-eFluorTM 710 (clone: SK3, eBioscienceTM), CD127 PE-Cy7 (clone: eBioRDR5, eBioscienceTM), and CD25 APC (clone: BC96, eBioscienceTM); the Foxp3 PE (clone: 235A/E7, eBioscienceTM) antibody was used for the detection of Treg signature transcription factor Foxp3. Representative gating strategy used for the data analysis is shown in Figure 1.

**Figure 2:**
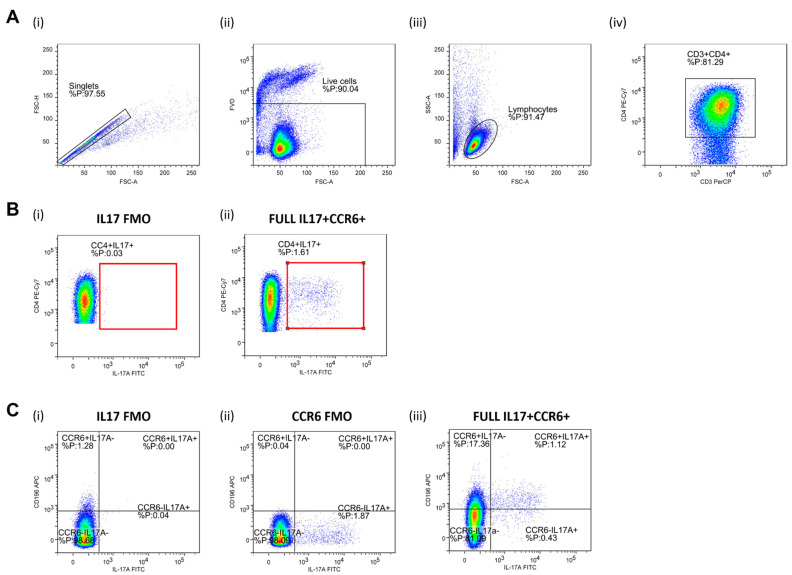
Gating strategy for the assessment of peripheral blood Th17 population by flow cytometry. Panel (**A**) shows representative dot plots illustrating gating strategy, including exclusion of doublets using forward scatter area (FSC-A) versus forward scatter width (FSC-W) analysis (**A**-**i**), gating on live cells negative for amine-reactive fixable viability dye (**A**-**ii**), lymphocytes (**A**-**iii**), CD3^+^ T cells (**A**-**iv**), and CD3^+^CD4^+^ T helper cells (**A**-**iv**). T helper cells were subsequently analyzed for IL-17A and CD196/CCR6 expression. First, total IL-17-secreting T helper cells were analyzed (Panel (**B**)) where the gate on IL-17^+^ T cells were defined using florescence minus one (FMO) control for IL17 antibody (**B**-**i**). A representative sample demonstrating relative frequencies of peripheral CD4^+^IL-17^+^ T helper cells in healthy young individuals are shown at (**B**-**ii**). The population of IL-17-secreting T helper cells was further analyzed for CD196/CCR6 expression (panel (**C**), **C-iii**), hence two subpopulations were identified—CD4^+^CD196^+^IL17^+^ corresponding to Th17 cells and CD4^+^CD196-IL17^+^ non-Th17 cells accounting for other T helper subpopulations with the capacity to secrete IL-17. FMO controls for antibodies with specificities for IL17 and CCR6 were utilized for the setup of quadrant gates (**C**-**i**, **C**-**ii**). FlowLogic software was used for data analysis and illustration.

Helper T Lymphocytes (Th17). CD4^+^ T cells were separated via negative magnetic selection (MagniSortTM Human CD4^+^ T cell, Enrichment kit; Invitrogen by Thermo Fisher Scientific, Waltham, MA, USA). Negatively selected cells were then stimulated for 4 h by phorbol 12-myristate 13-acetate (PMA) and ionomycin, in order to activate and promote cytokine production. CD4 T cell activation (short-term ~4 h) was carried out with a commercially available cell stimulation cocktail (500×; eBioscienceTM, Invitrogen by Thermo Fisher Scientific, Waltham, MA, USA), with an addition of calcium chloride (CaCl_2_) and brefeldin A solution (1000×; eBioscienceTM, Invitrogen by Thermo Fisher Scientific, Waltham, MA, USA) in 24-well plates under the following conditions: 4 h, ~37 °C, 5% CO_2_, > 80% humidity level). Activation was inhibited by adding 200 µL of 0.1 M EDTA to plates containing cells. For the detection of cell surface antigens, following the anti-body mixture, was used: CD3 PerCP-eFluorTM 710 (SK7, eBioscienceTM, Affymetrix by Thermo Fisher Scientific, CA, USA), CD4 PE-Cy7 (SK3, eBioscienceTM), CD196 APC (R6H1, eBioscienceTM), while RORɣt PE (AFKJS-9, eBioscienceTM), and IL-17A FITC (eBio64DEC17, eBioscienceTM) antibodies were added for the detection of intracellular antigens. Representative gating strategy used for the data analysis is shown in Figure 2.

### 2.9. Serum- and Glucocorticoid Regulated Kinase 1 (SGK1) Assay

Relative amount of total serum- and glucocorticoid regulated kinase 1 (SGK1) in cultured cells was measured by commercially available cell-based enzyme-linked immunosorbent assay (ELISA) kit (LifeSpan BioSciences, Inc., Seattle, WA, USA) on compact absorbance reader for 96-well microplates (BioRad PR 3100 TSC). After thawing and viability check (0.4% trypan blue solution), PBMCs were counted and seeded in culture media (RPMI-1640 supplemented with 10% FBS and 1% penicillin-streptomycin antibiotic). Prior to the addition of cells, 96-well plates were coated with 100 µL of adhesive poly-l-lysine solution (0.1% in H_2_O) and incubated for 30 min at 37 °C. Following incubation, 20–30,000 cells were seeded into each well in 200 µL culture media, and the plates were incubated overnight (~37 °C, 5% CO_2_, >80% humidity). Next, assay protocol steps provided in the kits user manual were followed. After completion of assay protocol, plates were read immediately at 450 nm, using the microplate reader (available at https://www.lsbio.com/elisakits/mouse-human-rat-phospho-sgk1-sgk-ser422-cell-based-phosphorylation-elisa-elisa-kit-ls-f1101/1101, accessed on 12 March 2021).

### 2.10. p38 Mitogen-Activated Protein (MAP) Kinase Assay

The relative amount of total and phosphorylated human p38 mitogen-activated protein kinase (p38/MAP kinase) was measured from cell lysates using a commercially available InstantOne ELISA Kit (Invitrogen by Thermo Fisher Scientific, Waltham, MA, USA). Previously stored PBMCs were thawed and stained with 0.4% trypan blue solution (Sigma-Aldrich, Merck KGaA, Darmstadt, Germany), in order to count viable cells in the Bürker-Türk chamber under a light microscope. The number of viable cells was adjusted at appropriate density of approximately 25,000 cells per well. Cells were resuspended in Hank’s Balanced Salt Solution (HBSS), containing 5% fetal bovine serum (FBS; Sigma Aldrich, St. Louis, MO, USA), and lysed with 20% final volume of lysis mix, provided in the kit. Following assay protocol steps, absorbance of samples was measured at 450 nm on compact absorbance reader for 96-well microplates (BioRad PR 3100 TSC, Bio-Rad Laboratories, Hercules, CA, USA).

### 2.11. Biomarkers of Oxidative Stress and Antioxidant Defense Assay: Thiobarbituric Acid Reactive Substances (TBARS), Ferric-Reducing Ability of Plasma (FRAP), and Serum 8-Iso Prostaglandin F2 (8-Iso-PGF2α) Protein Concentration

The thiobarbituric acid reactive substances (TBARS) method, which assess the lipid peroxidation products, presents a marker of oxidative stress level, while the ferric reducing ability of plasma (FRAP) method presents a marker of the antioxidant capacity of blood samples. Both TBARS and FRAP were measured using spectrophotometry, according to the protocol described, in detail, in an earlier paper of our research group [9]. The 8-iso-PGF2α serum concentration (product of the non-enzymatic peroxidation of arachidonic acid in membrane phospholipids) was measured using the commercially available ELISA kit (MyBioSource, MBS700957, San Diego, CA, USA).

### 2.12. Statistical Analysis

For normally distributed data, results were reported as the arithmetic mean and standard deviation (SD), while for non-normally distributed data results were reported as the median and interquartile. The Shapiro–Wilk normality test was used for the normality of data distribution assessment. When variables were normally distributed, differences between values measured before and after HS diet were tested by paired *t*-test. When variables were non-normally distributed, the Wilcoxon rank sum test was used. The correlations between normally distributed variables were tested by Pearson’s correlation test, and for non-normally distributed variables by Spearman’s correlation test. *p* < 0.05 was considered statistically significant. Statistical analysis was performed using SigmaPlot, version 11.2 (Systat Software, Inc., Chicago, IL, USA).

## 3. Results

All subjects were young, lean, normotensive, had normal renal function, full blood counts, serum electrolytes, and did not have an active inflammatory process (normal hsCRP value) (Table 1, LS column). They all completed two weeks’ dietary salt perturbation protocol, 7 days of LS diet (“wash-out” period), followed by 7 days of HS diet. Median salt intake on the last day of the 7-day run-in (LS) diet verified that the goal pursued by the “wash-out” period has been achieved (Table 1, LS column). A significant increase in 24-h urine sodium excretion, as well as in calculated daily salt intake, confirmed that participants conformed to the given LS and HS diet guidelines. There was no significant difference in 24-h urine total volume, creatinine coefficient, urea, protein, albumin, and potassium excretion, following the HS diet, compared to LS. As previously reported, the 7-day HS diet did not significantly change subjects’ BMI and WHR, compared to LS. Moreover, systolic BP, diastolic BP and mean arterial pressure (MAP) values remained unchanged, following the HS diet, compared to LS. Thus, since large changes in calculated daily salt intake and 24-h sodium excretion during HS intake were not accompanied with concomitant changes in BP level, all participants can be characterized as salt-resistant, and all the observed changes following HS diet reported in this manuscript can be considered as independent of BP. The HS diet significantly increased serum sodium and potassium concentration and decreased serum calcium concentration, compared to LS conditions; still, all the values were within normal reference range (Na 137–146 mmol/L, K 3.9–5.1 mmol/L, Ca 2.14–2.53 mmol/L). Interestingly, HS diet resulted in uniform reduction (but not statistically significant) of all blood cell types, compared to the LS conditions, which could potentially be the result of higher plasma volume, due to the HS diet, which is physiological presumably accompanied by increased thirst. There was no significant difference in serum creatinine, urea, and hsCRP levels, following the HS diet, compared to LS. Anthropometric, hemodynamic, and biochemical responses to a 7-day HS diet, in the study population, are presented in Table 1.

### 3.1. Serum Pro- and Anti-Inflammatory Cytokines, C3a Complement Component, Soluble Cell Adhesion Molecules, and Endoglin Protein Concentration

The serum protein concentration of pro-inflammatory cytokines IFNγ, TNF-α, IL-9, and IL-17A was significantly increased, while the concentration of IL-6 and IL-23 remained unchanged in the HS diet, compared to the LS diet. The serum protein concentration of anti-inflammatory cytokines IL-10 and TGF-β1 was significantly increased, while concentration of IL-21 and IL-22 remained unchanged, following the 7-day HS diet, compared to the LS diet. Serum protein concentration of C3a complement component, endoglin, and soluble cell adhesion molecules (sICAM-1, sVCAM-1, and E-selectin) was not significantly altered by dietary protocol (Table 2).

### 3.2. Frequencies of Regulatory T Lymphocytes (Treg) and Helper T Lymphocytes (Th17) and Th17/Treg Ratio

**Figure 3:**
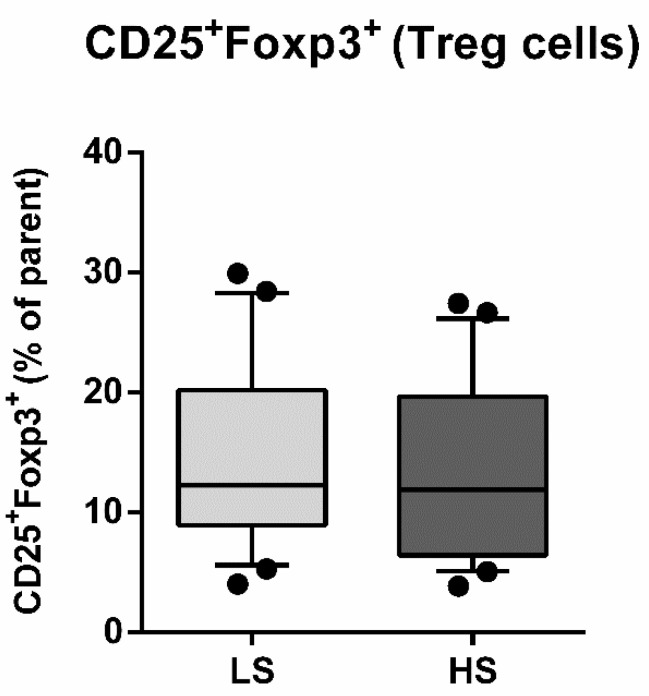
Effects of a 7-day high-salt diet on the frequency of peripheral regulatory T cells (Treg) in healthy young individuals. Relative frequencies of peripheral Treg are presented as box-and-whisker plots 10 to 90 percentiles; Paired *t*-test, LS vs. HS; *p* < 0.05 was considered significant.

**Figure 4:**
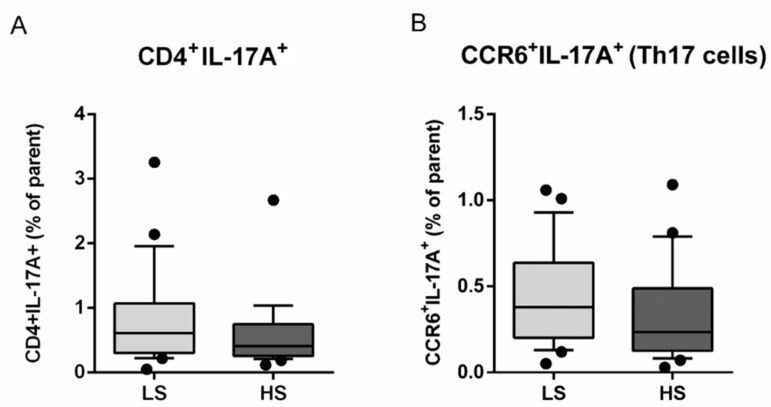
Effects of a 7-day high-salt diet on the representation of peripheral Th17 population in healthy young individuals. Relative frequencies of total IL-17-secreting peripheral T helper (CD3^+^CD4^+^) cells are shown at panel (**A**), while the relative frequencies of CCR6^+^IL17^+^ T cells, corresponding to Th17 cells, are demonstrated at Panel (**B**). Data are presented as box-and-whisker plots 10 to 90 percentiles. Paired *t*-test, LS vs. HS; *p* < 0.05 was considered significant.

The seven-day HS diet resulted in unchanged frequency of CD25/Foxp3-expressing peripheral blood lymphocytes (Treg), within the CD4^+^CD127^+^ subpopulation (CD25^+^Foxp3^+^% of parent LS 14.54 ± 7.76 vs. HS 13.49 ± 7.53, *p* = 0.288) (Figure 3). The frequency of total IL-17-secreting peripheral T helper cells was not significantly changed, following the HS diet (CD4^+^IL-17A^+^% of parent LS 0.80 ± 0.73 vs. HS 0.59 ± 0.53, *p* = 0.173) (Figure 4A). These cells were further immunephenotyped and, based on their CCR6 expression, subdivided to Th17 (CCR6^+^IL-17^+^) and non-Th17 (CCR6-IL-17^+^) T helper cells, with the latter corresponding mostly to IL-17-secreting Th1 and Th2 T helper cells. The HS diet did not induce significant change in the frequency of Th17 cells, compared to LS conditions (CCR6^+^IL-17A^+^% of parent LS 0.43 ± 0.28 vs. HS. 0.33 ± 0.28, *p* = 0.256) (Figure 4B). Th17/Treg ratio also remained unaltered, following HS diet, compared to LS conditions in healthy salt-resistant individuals (Th17/Treg LS 0.039 ± 0.036 vs. HS 0.031 ± 0.030, *p* = 0.347).

### 3.3. Serum- and Glucocorticoid Regulated Kinase 1 (SGK1) and p38 Mitogen-Activated Protein (MAP) Kinase

**Figure 5:**
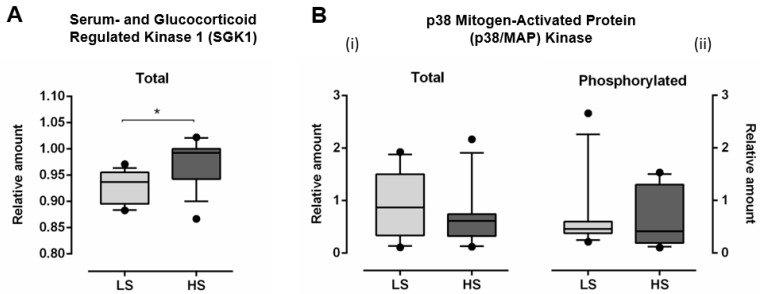
The effect of a 7-day high-salt diet on relative amount of total serum- and glucocorticoid regulated kinase 1 (SGK1) (**A**) and total (**B-i**) and phosphorylated (**B-ii**) p38 mitogen-activated protein (MAP) kinase (**B**) in young healthy individuals. *N* = 30 (15 women and 15 men). LS—low salt; HS—high-salt; N—number of subjects). Data are presented as box-and-whisker plots 10 to 90 percentiles. Paired *t*-test, * *p* < 0.05, LS diet vs. HS diet.

The relative amount of total SGK1 significantly increased (Total SGK1: LS 0.928 ± 0.031 vs. HS 0.975 ± 0.044, *p* < 0.001), following HS diet, compared to LS diet (Figure 5A). Relative amount of both total (Total p38/MAP kinase LS 0.887 ± 0.611 vs. HS 0.660 ± 0.549, *p* = 0.243) and phosphorylated p38/MAP kinase (Phosphorylated p38/MAP kinase LS 0.651 ± 0.677 vs. HS 0.639 ± 0.528, *p* = 0.947) was not significantly changed, following HS diet, compared to LS conditions (Figure 5B).

### 3.4. Markers of Oxidative Stress and Antioxidant Defense

TBARS levels (marker of lipid peroxidation) significantly increased, following the HS diet, compared to LS (Table 3). Similarly, 8-iso-PGF2α (marker of oxidative stress) serum protein concentrations significantly increased after the HS diet, compared to LS (Table 3). The 7-day HS diet significantly decreased FRAP levels (marker of antioxidant defense), following the HS diet, compared to LS (Table 3).

### 3.5. Correlations

There was moderate positive correlation between the 24-h urine natriuresis and IFNγ (r = 0.393, *p* = 0.047) and 24-h urine natriuresis and IL-9 (r = 0.445, *p* = 0.023). There was strong/moderate positive correlation between relative amount of phosphorylated p38/MAP kinase and sVCAM-1 (r = 0.621, *p* = 0.003) and endoglin (r = 0.456, *p* = 0.043) level. There was moderate positive correlation between the frequency of Th17 cells and IL-23 (r = 0.422, *p* = 0.032) levels.

There was only a moderate/strong negative correlation between the TBARS and Treg cell frequency (r = −0.424, *p* = 0.008), while FRAP strongly negatively correlated with 24-h natriuresis alone (r = −0.558, *p* < 0.001). There was no significant correlation between measured biomarkers of oxidative stress and pro-/anti-inflammatory cytokines level, markers of endothelial activation, or the frequency of peripheral Th17 lymphocytes and enzymes involved in its activation and differentiation (SGK1 and p38/MAP kinase).

## 4. Discussion

The salient findings of the present study are that the 7-day HS diet, in healthy salt-resistant individuals, promotes pro-inflammatory response, characterized by an increase in serum levels of IFNγ, TNF-α, IL-9, and IL-17A, but also results in an opposing rise in anti-inflammatory IL-10 and TGF-β1 levels. The increase in the serum IL-17A level (and trend of increase in IL-21 and IL-22 level), as well as the increase in a relative amount of total SGK1 in PBMCs, supports the hypothesis of initial activation of IL-17-secreting Th17 cells, in response to the HS diet. Interestingly, the HS diet triggers a concomitant anti-inflammatory response, potentially mediated by Treg cells activation and characterized by an increase in serum concentration of IL-10 and TGF-β levels. The present study showed that oxidative stress alters immune regulatory mechanisms, due to HS loading potentially via effects on Treg cells.

### 4.1. Salt Intake and Modulation of Early Inflammation

Studies in animal models consistently indicate that HS intake stimulates the production of several cytokines known to promote inflammation and organ damage. However, data on clinical studies in healthy volunteers are scarce. Results of the present study demonstrate an increase in pro-inflammatory cytokines (IFNγ, TNF-α, IL-9, and IL-17A) and total SGK1 level, following HS diet, which is similar to previously described findings in animals. For example, HS diet induced an increase in IL-6 level in the kidney of Dahl salt-sensitive rats, which was associated with the development of high BP and organ damage [25]. The HS diet increased the production of TNF-α in mouse macrophages [26], and increased peritoneal, aorta, and para-aortic tissue IL-6 and SGK-1 mRNA expression in nephrectomized rats [27]. The HS-fed mice presented with dysfunctional Treg lymphocytes producing significantly higher IFNγ compared to mice fed with normal salt [28]. In the present study, the HS diet significantly increased serum level of pro-inflammatory cytokines (TNF-α, IFNγ, IL-9, and IL-17A). In addition, there was moderate positive correlation between salt intake and IFNγ, as well as between salt intake and IL-9 level, indicating an association between salt loading and the activation of pro-inflammatory response.

Beside cardiovascular diseases, including hypertension, have been recently characterized with an imbalance between Th17 and Treg cells [20], and excessive salt intake/exposure has been shown as a novel and potent driver of Th17 cell differentiation and autoimmunity [29,30,31]. A particularly important cytokine in the development of cardiovascular diseases is IL-17A, which is mainly produced by Th17 cells. It has been demonstrated that IL-23 reinforces the Th17 phenotype by increasing the expression of IL-23 receptor (IL-23R), resulting in pathogenicity of Th17 cells, but it also suppresses development of Treg cells [16,32,33]. On the other hand, Treg cells (FoxP3^+^), with TGF-β as a critical regulator of its development and function and its key product, anti-inflammatory cytokine IL-10, are involved in immune tolerance and homeostasis [34,35]. In the present study, the short-term HS diet resulted in increased level of IL-17A, indicating the activation of Th17 cells, as well as in moderate positive correlation between IL-23 serum level and frequencies of Th17 cells, supporting IL-23/Th17 cells axis in Th17 cells activation. Interestingly, TGF-β was also significantly increased with the HS diet in the present study, suggesting that it may be a key player in simultaneous activation of anti-inflammatory hand of Th17/Treg activation in response to HS diet.

Further support for this hypothesis is the finding of significantly increased levels of SGK1 after the HS diet. SGK1, which is downstream of IL-23R and p38/MAPK-dependent pathway, is a critical factor that reciprocally regulates development of Th17/Treg balance [32,36,37,38]. SGK1 regulates IL-23R expression, important for the differentiation of Th17 cells [16], but also negatively regulates Treg development and function (e.g., Treg-suppressive function increased after the loss of SGK1in Treg cells) [32]. It has been demonstrated that excessive SGK1 activity, stimulated by oral NaCl and renal NaCl retention, could lead to development and progression of arterial hypertension [39]. Additionally, oxidative stress, which is known to be significantly increased in HS conditions [9], has been shown as the most powerful specific stress activating p38/MAP kinase [40]. Taken together, present results support the important role of SGK1 in regulating immune responses due to HS loading.

Although the results of animal studies imply involvement of Th17/Treg imbalance in the inflammation initiation and final organ damage induced by HS intake [15,16,28], there are a limited number of clinical studies investigating this issue. For example, HS diet-induced Th17 generation increased severity of experimental autoimmune encephalomyelitis, suggesting the potential clinical relevance of salt intake in Th17-associated autoimmune disease [17]. Even though in the present study frequency of Th17 cells was not significantly changed following the HS diet, a significant increase in IL-17A serum level and total SGK1 in PBMCs suggest Th17 cells activation. A recently published pilot study demonstrated that a 14-day HS diet significantly increased Th17 cells frequency in PBMCs of healthy man volunteers [41]. Luo et al. reported significant increase in Th17/Treg ratio during a 7-day HS diet, as well as its decrease during a 7-day LS diet in healthy volunteers [20]. On the other hand, a cross-sectional pilot study showed that the degree of salt intake (LS ≤ 5 g salt/day vs. HS ≥ 5 g salt/day) is not significantly associated with the proportion of Treg and Th17 lymphocytes in the PBMCs from healthy subjects [42].

**Figure 6:**
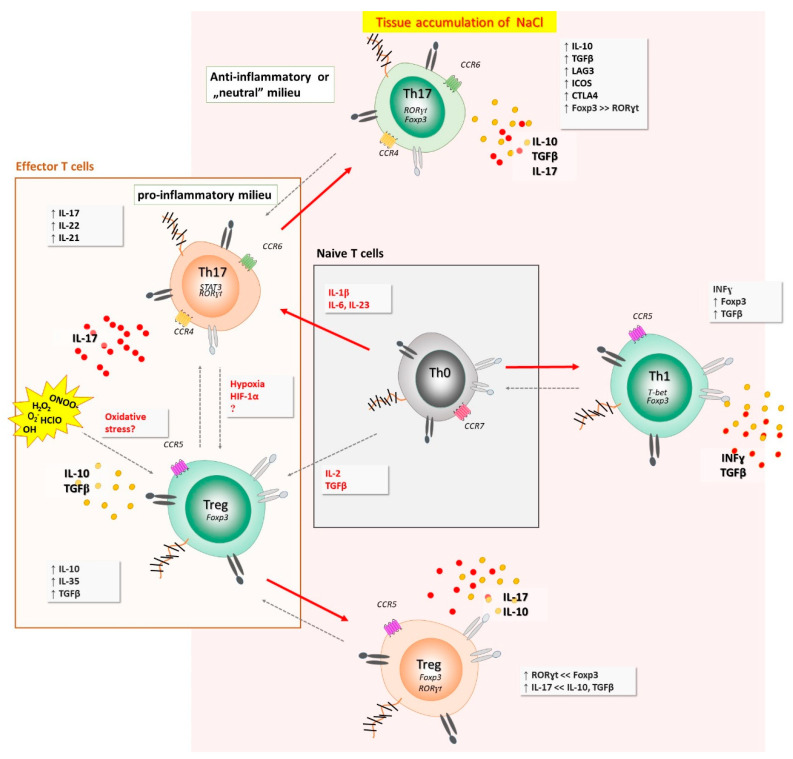
Dichotomous, context-dependent fate of T helper cells, when exposed to high-salt conditions. Naïve cell, exposed to hyperosmotic and pro-inflammatory environment, primed under Th17 polarizing cytokines, acquired enhanced Th17 function. This effect was also observed among other T helper cell compartments (i.e., Th1 subpopulation). Interestingly, if the Th17 cells are void of pro-inflammatory milieu during activation in high NaCl environment (and re-activation at the sites of ongoing immune responses against certain pathogens or (auto)antigens), they acquire anti-inflammatory phenotype and function, characterized by the up-regulation of IL-10, TGFβ, Foxp3, CTLA-4, ICOS, and LAG3. Increased oxidative stress may alter immune regulatory mechanisms, potentially via effects on Treg cells.

Although the HS diet elicits pro-inflammatory response, there are novel findings suggesting that a short-term HS diet may exert the opposite effects in salt-resistant conditions. In one hand, exposure of cultured T cells to a HS strongly increased IL-17A and IL-22 expression in Th17 cells, but surprisingly, also upregulated FoxP3, IL-10, and TGF-β and reduced IFNγ co-expression, indicating anti-inflammatory Th17 cell phenotype [43]. The presence of pro-inflammatory cytokines (IL-6, IL-1β, IL-23, and IL-21) abrogated HS-induced anti-inflammatory response and gave rise to a pro-inflammatory Th17 cell phenotype. Taken together, these findings, for the first time, revealed a context-dependent, dichotomous role of HS in modulating Th17 cell pathogenicity and indicated a dominant role of pro-inflammatory cytokines over potential anti-inflammatory effects that NaCl directly exerts on effector T cells [43]. All these could potentially explain at least a part of the results obtained in the present study, i.e., besides the expected pro-inflammatory Th17 cells-mediated response and increase in pro-inflammatory cytokines level due to HS intake, there is also HS-induced anti-inflammatory effect involving mechanisms (anti-inflammatory Th17 cells or some other), which are yet to be investigated and clarified in further clinical studies. Such dichotomous context-dependent fate of T helper cells, when exposed to high-salt conditions, are summarized in Figure 6.

Importantly, the immune mechanisms related to the regulation of BP are quite different between salt-sensitive (SS) and salt-resistant (SR) rats [44]. For example, high-fructose intake differentially affects SS and SR rats in such a way that, in SS rats, it induces hypertension by increasing Th17 cells, which secrete pro-inflammatory cytokine CD-17A and involves IL-23R-SGK1 axis, but SR rats seems to be protected by producing a large amount of anti-inflammatory cytokine IL-10, secreted by Treg cells [44]. Interestingly, we find these results were very consistent with the results of the present study, suggesting that activation of anti-inflammatory responses, mediated by Tregs, due to short-time HS intake, could counteract the Th17 pro-inflammatory function and act as protective mechanism from BP increase and endothelial activation, at least to a certain point in healthy salt-resistant individuals.

### 4.2. Salt Intake and Endothelial Activation

CAMs, such as ICAM-1, VCAM-1, and E-selectin, as well as endoglin (which is endothelial co-receptor for several ligands of the TGF-β family), have been emerged as important markers of endothelial activation, preceding the adhesion of the activated leukocytes and initiating atherosclerotic lesions [45,46]. Available data on the effect of HS diet on markers of endothelial activation is limited, especially in healthy individuals. For example, plasma sE-selectin, but not sICAM-1 and sVCAM-1, levels increased within 2 weeks of the HS diet in salt-sensitive patients [18]. Furthermore, Tadzic et al. reported that, in hypertension, there is high concentration of sICAM-1 and sVCAM-1, with low sE-selectin levels, as markers of endothelial cell activation [47]. Earlier study of our research group showed that, even though microvascular reactivity, in response to vascular occlusion, was impaired following the 7-day HS diet, serum sCAMs (sICAM-1, sVCAM-1, and E-selectin) were unaffected by the HS diet protocol in young healthy women [48]. Finding that hypoxia-induced p38/MAP kinase activation increased endoglin expression in mouse brain microvascular endothelial cells [49], suggests an important role of p38/MAP kinase and its axis (involving SGK1) in the process of endothelial activation in response to various stress stimuli. This finding is also supported by the significant increase in TGF-β, which is a ligand for endoglin, and positive correlation between endoglin and relative amount of phosphorylated p38/MAP kinase level, following the HS diet observed in the present study. Our results are further supported by the fact that TGF-β induces the differentiation of CD4^+^ T cells into Th17 cells and Treg cells, thus controlling the immune response even further [50]. Still, whether the duration of HS loading, amount of taken salt, health condition, age, salt resistance status, or activation of protecting anti-inflammatory response, besides low-grade inflammation, following HS loading in healthy individuals, lies in the background of the obtained results remains to be elucidated.

### 4.3. Increased Oxidative Stress Due to High-Salt Intake and Early Inflammation

Earlier studies in both animals and humans (healthy and cardiovascular patients) repeatedly demonstrated the central role of increased level of oxidative stress in pathophysiological pathway resulting in impaired endothelial function, due to HS intake [8,51,52]. This is supported by the results of experiments which directly measured biomarkers of oxidative stress level and antioxidant defense (e.g., O_2_-production, antioxidant enzymes, TBARS, FRAP, 8-iso-PGF2α, intracellular oxidative stress, etc.), following the HS diet, as well as those which yielded functional evidence. These experiments demonstrated that increase in antioxidant capacity during HS loading (e.g., supplementation of antioxidant vitamins C and E) prevents development of endothelial dysfunction caused by HS intake [9]. This is in line with the studies reporting that the ability of a cell to counteract stressful conditions (as HS loading) requires the activation of cellular pathways conferring protection against oxidative stress, among which a key role is played by activation of so-called vitagenes (genes encoding heat shock proteins, thioredoxin/thioredoxin reductase system) [53,54,55]. The question of whether maintaining the oxidative balance during HS loading (e.g., by vitamins C and E supplementation) prevents its deleterious effects (endothelial dysfunction, early inflammation), potentially by activating vitagenes, opens a new and challenging area for future research related to the HS intake. Discovery of novel and potent inducers of vitagenes is expected to facilitate the development of pharmacological strategies to increase the intrinsic capacity of minimizing oxidative damage in various pathological states and diseases induced by oxidative stress [53,54,55]. This study confirmed that a 7-day HS diet results in increased lipid peroxidation and the consequent production of 8-iso-PGF2α (product of non-enzymatic ROS-induced peroxidation of arachidonic acid), as well as decreased antioxidant defense in healthy individuals [9]. Yet, the novelty of present study is the existence of a negative association between lipid peroxidation and peripheral Treg cells frequency, following the HS diet, suggesting that changes in oxidative balance following HS diet may alter immune regulatory mechanisms, possibly those mediated by Treg cells. This is a step further in understanding the link between oxidative stress and increased vascular inflammation, leading to endothelial dysfunction, which underlies all cardiometabolic diseases.

Study limitations. The present study involved female participants, engaged to the study protocol in different phases of menstrual cycle; however, the exact phase of menstrual cycle, in which each participant entered the study protocol, has not been assessed, what can be considered as potential limitation of this study.

## 5. Conclusions

In conclusion, in present study, increased levels of pro-inflammatory cytokines (TNF-α, IFNγ, IL-9, and IL-17A), but also of anti-inflammatory cytokines (TGF-β1 and IL-10), following the HS diet, indicate that HS diet induces an early pro-inflammatory, but also induces counterbalancing anti-inflammatory responses in young salt-resistant normotensive individuals. This is supported by positive correlation between salt intake and level of pro-inflammatory cytokines in this population. Positive correlation between the IL-23 level and frequency of Th17 cells, as well as increased level of IL-17A and SGK1, in conditions of HS loading, support the role of IL-23/SGK1/Th17 cells/IL-17A axis in immune response due to HS loading in healthy individuals. Positive correlation between relative amount of phosphorylated p38/MAP kinase and markers of endothelial activation (sVCAM-1 and endoglin) indicates potential role of p38/MAP kinase axis in the resulting endothelial activation due to HS intake. Taken together, a short-term HS diet promotes low-grade inflammation, characterized by the early activation of Th17 cells. Additionally, there is protective anti-inflammatory response of Treg cells to HS loading (possibly via TGF-β pathway), which may protect salt-resistant individuals from more prominent endothelial activation and dysfunction. Results suggest the possibility that oxidative stress alters immune regulatory mechanisms via effects on Treg cells, which remains to be investigated.

## Figures and Tables

**Table 1:** Anthropometric, hemodynamic, and biochemical responses to a 7-Day high-salt diet in young healthy individuals.

Parameter	LS	HS
N (F/M)	30 (15/15)	
Age (Years)	21 [19–23]	
Anthropometric Parameters		
BMI (kg/m^2^)	24.03 [22.14–25.91]	24.03 [22.46–25.91]
WHR	0.83 [0.76–0.87]	0.82 [0.77–0.84]
Hemodynamic Parameters		
SBP (mmHg)	116 ± 11	116 ± 13
DBP (mmHg)	75 [70–80]	73 [71–78]
MAP (mmHg)	88 ± 7	88 ± 6
24-h Urine Biochemical Parameters		
24 h urine volume (mL)	1573 ± 760	1645 ± 740
24 h creatinine coefficient (µmol/24 h/kg)	153.5 ± 62.6	157.0 ± 52.5
24 h urine urea (mmol/dU)	234.3 ± 132.1	238.5 ± 100.6
24 h urine protein (mg/dU)	91.5 [50.0–107.8]	81.0 [66.0–107.5]
24 h urine albumin (mg/dU)	5.3 [2.5–9.4]	5.6 [4.0–8.0]
24 h sodium (mmol/dU)	66.0 [46.0–114.8]	211.5 [162.5–336.0] *
24 h potassium (mmol/dU)	40.5 ± 17.1	48.8 ± 23.3
calculated salt intake (g/day)	3.9 [2.7–6.7]	12.4 [9.5–19.6] *
Serum Biochemical Parameters		
leukocytes (×10^−9^/L)	6.2 [5.5–7.4]	6.0 [5.2–7.3]
erythrocytes (×10^−12^/L)	5.02 ± 0.55	4.89 ± 0.55
hemoglobin (g/L)	145.3 ± 15.7	142.0 ± 15.7
hematocrit (%)	42.2 ± 4.4	41.4 ± 4.3
thrombocytes (×10^−9^/L)	242.3 ± 38.4	229.1 ± 42.3
urea (mmol/L)	4.3 ± 1.1	4.4 ± 0.9
creatinine (µmol/L)	76.7 ± 12.6	71.1 ± 9.4
sodium (mmol/L)	138.0 [137.0–139.0]	140.0 [139.0–141.0] *
potassium (mmol/L)	4.0 [3.9–4.2]	4.1 [4.0–4.3] *
calcium (mmol/L)	2.48 ± 0.07	2.43 ± 0.10 *
hsCRP (mg/L)	0.55 [0.32–1.13]	0.56 [0.34–0.80]

Data are presented as the mean ± standard deviation (SD) (normally distributed data) or median and interquartile range (non-normally distributed data). LS—low salt; HS—high-salt; N—number of participants; F—female; M—male; BMI—body mass index; WHR—waist-to-hip ratio; SBP—systolic blood pressure; DBP—diastolic blood pressure; MAP—mean arterial pressure; hsCRP—high-sensitivity C reactive protein. * *p* < 0.05 LS vs. HS (Paired *t*-test).

**Table 2:** Serum protein concentration of pro- and anti-inflammatory cytokines, C3a complement component, soluble cell adhesion molecules, and endoglin responses to a 7-day high-salt diet in healthy young individuals.

Parameter (pg/mL)	LS	HS
N (F/M)	30 (15/15)	
Pro-inflammatory cytokines		
IFNγ	1.26 [0.76–1.58]	1.80 [1.31–4.85] *
TNF-α	7.55 ± 12.27	14.49 ± 16.23 *
IL-6	2.24 [1.35–3.18]	2.82 [1.93–3.53]
IL-9	0.09 [0.05–0.23]	0.19 [0.11–3.49] *
IL-17A	1.52 [0.89–4.67]	2.02 [1.56–12.2] *
IL-23	0.05 ± 0.02	0.05 ± 0.02
Anti-inflammatory (immunomodulatory) cytokines		
IL-10	0.44 [0.27–0.66]	0.68 [0.44–3.60] *
IL-21	168.2 ± 210.5	281.6 ± 296.8
IL-22	1.39 [1.10–2.18]	1.88 [1.29–3.21]
TGF-β1	36.6 ± 33.0	49.7 ± 31.6 *
C3a Complement Component	202.3 ± 159.4	204.5 ± 178.0
Soluble Cell Adhesion Molecules		
sICAM-1	1108 [444–1623]	968 [537–1558]
sVCAM-1	3342 [2195–4808]	3230 [2524–4024]
E-selectin (CD-62E)	132.4 ± 47.5	130.5 ± 39.3
Endoglin	1753 [1118–2679]	1709 [1337–2599]

Data are presented as the mean ± standard deviation (SD) (normally distributed data) or median and interquartile range (non-normally distributed data). LS—low salt; HS—high-salt; N—number of subjects; F—female; M—male; IFNγ—interferon gamma; TNF-α—tumor necrosis factor alpha; IL-6—interleukin 6; IL-9—interleukin 9; IL-17A—interleukin 17A; IL-23—interleukin 23; IL-10—interleukin 10; IL-21; interleukin 21; IL-22—interleukin 22; TGF-β1—transforming growth factor beta; cICAM-1—soluble intercellular adhesion molecule 1; sVCAM-1—soluble vascular cell adhesion molecule 1. * *p* < 0.05 LS vs. HS (Paired *t*-test or Wilcoxon signed-rank test).

**Table 3:** Thiobarbituric acid reactive substances (TBARS), ferric-reducing ability of plasma (FRAP), and serum 8-iso prostaglandin F2 (8-iso-PGF2α) protein concentration responses to a 7-day high-salt diet in healthy young individuals.

Parameter (pg/mL)	LS	HS
N (F/M)	30 (15/15)	
Biomarkers of Oxidative Stress Level		
TBARS (μM/MDA)	21.6 [16.8–24.1]	24.3 [19.5–26.4] *
8-iso-PGF2α (pg/mL)	692.5 ± 112.3	813.7 ± 78.7 †
Biomarker of Antioxidant Defense		
FRAP (mM/L TE)	0.33 [0.30–0.39]	0.21 [0.18–0.28] ‡

Data are presented as the mean ± standard deviation (SD) (normally distributed data) or median and interquartile range (non-normally distributed data). LS—low salt; HS—high-salt; N—number of subjects; F—female; M—male; TBARS—Thiobarbituric Acid Reactive Substances; FRAP—Ferric-Reducing Ability of Plasma; 8-iso-PGF2α—8-iso Prostaglandin F2α. * *p* < 0.001, † *p* < 0.001, ‡ *p* < 0.001 LS vs. HS (paired *t*-test).

## Data Availability

All of the data is contained within the article.
